# Using Class-Specific Feature Selection for Cancer Detection with Gene Expression Profile Data of Platelets

**DOI:** 10.3390/s20051528

**Published:** 2020-03-10

**Authors:** Lei-ming Yuan, Yiye Sun, Guangzao Huang

**Affiliations:** 1 College of Electrical & Electronic Engineering, Wenzhou University, Wenzhou 325035, China; yuan@wzu.edu.cn; 2 Department of Planning & Finance, Wenzhou University, Wenzhou 325035, China; 20170135@wzu.edu.cn

**Keywords:** cancer detection, multi-class classification, platelets, elastic net, class-specific features, probabilistic support vector machine

## Abstract

A novel multi-classification method, which integrates the elastic net and probabilistic support vector machine, was proposed to solve this problem in cancer detection with gene expression profile data of platelets, whose problems mainly are a kind of multi-class classification problem with high dimension, small samples, and collinear data. The strategy of one-against-all (OVA) was employed to decompose the multi-classification problem into a series of binary classification problems. The elastic net was used to select class-specific features for the binary classification problems, and the probabilistic support vector machine was used to make the outputs of the binary classifiers with class-specific features comparable. Simulation data and gene expression profile data were intended to verify the effectiveness of the proposed method. Results indicate that the proposed method can automatically select class-specific features and obtain better performance of classification than that of the conventional multi-class classification methods, which are mainly based on global feature selection methods. This study indicates the proposed method is suitable for general multi-classification problems featured with high-dimension, small samples, and collinear data.

## 1. Introduction

Cancer detection plays a vital role in improving the survival rate and living quality of patients. Methods of detecting cancer mainly include clinical symptom analysis, pathological analysis of cancer tissue, radiology, biochemical examination and molecular diagnosis, etc. At present, using the approach to detect the gene expression profile data has emerged as a potential method for detecting cancer. For example, it is reported that the gene expression profile data of platelets can be used to detect cancer [1,2]. Platelets are the primary cell type of blood and known for their hemostatic property. Platelets play a crucial role in the process of tumor growth and metastasis, thereby altering their gene expression profile in cancer patients [1,2]. It shows that the platelets may be used as an all-in-one bio-source for the diagnosis of different types of cancer and precancerous tumors. Using the gene expression profile data of platelets to detect cancer has the unique advantage of micro-invasive molecular diagnostics, overcoming the difficulty of obtaining tissues [3].

Cancers are complex diseases, and are related to many etiological factors, especially in the early stage of cancer development [4,5]. It is hard to observe the expression levels of several marker genes for effective cancer diagnosis. Therefore, classification methods via machine learning algorithms are used to detect cancer with the gene expression profile data [6,7]. However, the gene expression profile data encompasses the expression levels of many genes assayed in relatively few samples. This is also called the “large p, small n” problem [8,9,10,11], which has been a difficulty in the field of machine learning. For the expression profile data with the “large p, small n” problem, feature selection has a direct effect on the generalization and the training efficiency of the classifier. The conventional methods of feature selection, such as Relieff and support vector machine recursive feature elimination (SVM-RFE), apply either single-variable ranking or recursive variable selection to reduce the number of features that are finally substituted into the classifier [12,13,14,15,16,17,18,19]. As a regularized regression method with automatic feature selection, the elastic net [20,21] naturally mitigates the “large *p*, small *n*” problem. Besides, the gene expression profile data is collinear as multiple genes tend to function together, e.g., in the shared biological pathways, and thus the correlations in expression level among those genes can be high. As for collinear data, the elastic net is able to select groups of features, rather than individual features, which are highly correlated.

In the field of machine learning, researches on the feature selection method for the multi-classification problem is mostly based on global features. It is actually the same case for cancer detection with gene expression profile data. In addition to the global feature selection method, there is another way to select features for the multi-classification problem, which is the class-specific feature selection method. There are few studies investigating on solving multi-class classification problems from the angle of the class-specific feature selection method. The class-specific feature selection method is related to the one-vs-all (OVA) strategy [22], which is commonly used in the multi-class classification problem. With the OVA strategy, the multi-class classification problem is decomposed into multiple binary classification problems, whose features can be optimized individually, and the selected features are called the class-specific features. Therefore, the class-specific features for these obtained binary classification problems are different, and increases the optimization space of the classification algorithm relative to the one based on the global features. Besides, the multi-class classification algorithm proposed on the basis of class-specific features is more complex than that based on the global features, and that how to fuse the outputs of the binary classifiers based on the class-specific features to get the final classification result of the initial multi-classification problem is an ongoing concern.

In this study, it was focused on the problem of how to use the class-specific feature selection method to enhance the performance of the classification algorithm in cancer detection with gene expression profile data. In this work, it was presented a new multi-class classification algorithm, which integrates the elastic net and probabilistic support vector machine. The OVA strategy was used to decompose the multi-class classification problem of cancer detection with gene expression profile data into multiple binary classification problems. The elastic net was conducted to select class-specific features for the obtained binary classification problems, on consideration of the characteristics of the elastic net and gene expression profile data. Besides, the probabilistic support vector machine was employed to construct the binary classifiers with the selected class-specific features. The probabilistic support vector machine inherits advantages of support vector machine (SVM), such as excellent generalization ability for the problem with nonlinear, small sample and high dimension. The probabilistic support vector machine converts the decision output of SVM into the form of probability, which makes the outputs of the binary classifiers based on the class-specific features comparable. The simulation data and gene expression profile data were obtained to verify the effectiveness of the proposed method and the results were compared with the conventional methods, such as SVM classifier combined with global feature selection methods, including Relieff and SVM-RFE [23,24,25].

### Statement of Societal Impact

The blood-based liquid biopsies belong to a noninvasive cancer detection technology receiving increasing attention nowadays. The blood plate is an excellent biomarker among various biomarker sources for blood-based liquid biopsies. The mRNA profiles of tumor-educated blood plates have been used to train classifiers to detect cancers. This paper proposed a new idea that embedding elastic net in classifiers for solving the problem of detecting cancer using tumor-educated platelets dataset. We adopt classifiers of SVM and probabilistic SVM to implement this idea, named as ESVM and EPSVM respectively.

The proposed classifiers are applied to detection cancer with the transcriptomic data derived from tumor-educated platelets from individuals. The experiment results show that the proposed methods have the advantage of automatically selecting the feature subsets to improve the generalization ability of classifiers comparing to the conventional multi-classification methods. 

The rest of the context is organized as follows: the related theories are described in Section 2. Original contributions are introduced in Section 3. The experimental procedures are presented in Section 4. Section 5 covers the results and discussion, and Section 6 gives the conclusion.

## 2. Related theories

### 2.1. Support Vector Machine and Probabilistic Support Vector Machine

SVM [26,27,28] is an excellent machine learning algorithm based on the statistical learning theory. A separating hyperplane was constructed from a low-dimensional space by SVM classifier as Equation (1) shown:

(1) w⋅x+b=0.

In doing that, it seeks the support vectors to support the hyperplane, using training data $D={\left\{x_{i},y_{i}\right\}}_{i=1}^{n}$, where $x_{i}\in R^{p}$ is a feature vector and $y_{i}\in \left\{1,-1\right\}$ is its class label. It is a constrained minimization problem, where w and b can be optimized by the Lagrange approach. The class label is predicted as Equation (2) shown

(2) $$y=\mathrm{sgn}\left(f\left(x\right)\right)=\mathrm{sgn}\left(\sum_{i=1}^{n}y_{i}a_{i}K\left(x_{i},x\right)+b\right),$$

where $a_{i}$ is Lagrange multiplier and $K\left(x_{i},x\right)$ is a kernel function. Using kernel tricks, SVM essentially transforms the linearly non-separable problems in the low-dimensional space into linearly separable ones in the high-dimensional space.

However, the standard SVM classifier does not provide such probabilistic output. To address this need, the output of the SVM classifier has been calibrated into a posterior probability. Here, a typical probabilistic SVM algorithm (PSVM) was introduced in briefly [29,30].

With the data $D={\left\{x_{i},y_{i}\right\}}_{i=1}^{n}$, signals of +1 and $N_{+}$ denote the label and number of positive samples, and signals of −1 and $N_{-}$ denote the label and number of negative samples respectively. The decision output of SVM is converted into the posterior probability by a sigmoid function as Equation (3) shown.

(3) $$P\left(y=1|x\right)\approx P_{A,B}\left(y=1|f\left(x\right)\right)=\frac{1}{1+\exp\left(Af\left(x\right)+B\right)},$$

where f(x) is the decision output of SVM classifier given in Equation (2). The parameters A and B are determined in Equation (4) by minimizing the negative log-likelihood problem

(4) $$\min\;F\left(A,\;B\right)=-\sum_{i=1}^{n}t_{i}\log\left(p_{i}\right)+\left(1-t_{i}\right)\log\left(1-p_{i}\right)$$

where,

(5) $$p_{i}=P_{A,B}\left(f\left(x_{i}\right)\right),\;\mathrm{and}\;t_{i}=\{\begin{array}{c} \frac{N_{+}+1}{N_{+}+2},\begin{array}{c} \mathrm{if}\begin{array}{c} y_{i}=1 \end{array} \end{array}\\ \frac{1}{N_{-}+2},\mathrm{if}\begin{array}{c} y_{i}=-1 \end{array} \end{array},i=1,\cdots ,n.$$

### 2.2. Feature Selection Methods

The feature selection methods can be divided into two categories: filter algorithms and wrap algorithms [12,13,14,31]. The filter algorithms are independent of the specific classification algorithm and directly use the statistical characteristics of data to select features. The advantage of filter algorithms is a high efficiency, but the shortcoming is not effective in removing redundant features. The wrap algorithms are based on classifiers and take the performance of classifiers as the evaluation index of selected features. The wrap algorithms have the advantage of high accuracy, but a drawback of large computation costs for training classifiers repeatedly.

#### 2.2.1. Relieff Algorithm

Relieff [32,33] is a classic filter algorithm. Relieff randomly selects a sample R from the training set and then searches K nearest neighbors from the same class, called near hits and K nearest neighbors form the different classes, called near misses. For a feature, if R is closer to the near-hits than the near misses, then this feature is beneficial to distinguish R from the different classes and the weight of this feature should be increased. Conversely, the weight of this feature should be reduced. This process is repeated several times to get the average weight of this feature. According to the weights of all features, Relieff ranks the importance of all features from most to least significant.

#### 2.2.2. SVM-RFE

SVM-RFE [34] is a representative of wrap algorithms. SVM-RFE constructs SVM classifiers through the training sets. The performance of the SVM classifier is related to the weight vector W. Therefore, SVM-RFE ranks the importance of features according to their impacts on the weight vector W. SVM-RFE assumes that all features are important in the initial run and then removes one feature with the lowest ranking coefficient by the way of iteration. After removing all features one by one, SVM-REF gives a ranking of the importance of all features from most to least significant.

#### 2.2.3. Elastic Net

Let $Y={\left[y_{1},\cdots ,y_{n}\right]}^{T}$ denote the response vector, and $X=\left[x_{1};\cdots ;x_{n}\right]$ denote the feature matrix. The objective function of the elastic net is given by:

(6) $$\hat{\beta }=\mathrm{ARG}\min\left\{{\left\|Y-X\beta \right\|}^{2}+\lambda \left[\alpha {\left|\beta \right|}_{1}+\left(1-\alpha \right){\left\|\beta \right\|}^{2}\right]\right\},$$

where λ>0 controls the amount of shrinkage and α∈(0,1) controls the balance between the L1 penalty term and the L2 penalty term [20].

With α=1, Equation (6) corresponds to the least absolute shrinkage and selection operator (LASSO) [35,36], which uses the L1 norm as the penalty term. With the natural attribute of the L1 penalty term, LASSO can shrink some of the estimated coefficients exactly to zero. Therefore, we can automatically select the features with non-zero regression coefficients as informative ones. However, for the “large p, small n” problem, LASSO would only select at most n features. Besides, LASSO tends to select only one feature among these features that are highly correlated. Therefore, for the gene expression profile data with problems of high dimension, small sample, and collinearity, LASSO is not an effective feature selection method. With α=0, Equation (6) corresponds to ridge regression [37], which uses the L2 norm as the penalty term. Ridge regression has the advantage in estimating coefficients for collinear data, but it cannot really shrink estimated coefficients to zero. The elastic net uses a hybrid penalty term of the L1 and L2 norms. It is known that the elastic net outperforms LASSO method in the highly correlated data with the “large p, small n” problem.

Relieff and SVM-RFE are based on ranking methods, which produce a series of candidate nested feature subsets according to the threshold values. The elastic net is based on the sparse attribute of the L1 penalty term, which can form a set of candidate feature subset directly according to the values of λ. Therefore, the elastic net is more flexible than Relieff and SVM-RFE in creating the candidate feature subsets.

## 3. The Proposed Method of Embedding Elastic Net in PSVM

**Figure 1:**
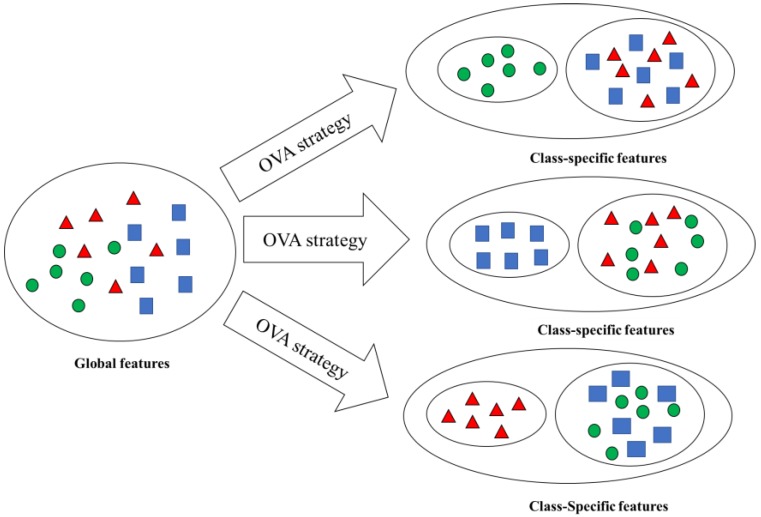
The idea of solving the multi-class classification problem using class-specific features by one-against-all (OVA) strategy.

The multi-class classification problems can be decomposed into a series of binary classification problems by OVA strategy, where one class is treated as the positive class and the rest classes are treated as the negative class. These binary classification problems are solved with global features in general. However, for a special positive class, the global features may not be the best class-specific features. If the class-specific features of positive classes are similar, then using the global features are suitable for these binary classification problems. However, if the class-specific features of positive classes are very different from one another, then using class-specific features to solve these binary classification problems will achieve better results than using global features. The idea of solving the multi-class classification problems was demonstrated by using class-specific features by the OVA strategy in Figure 1.

As shown in Figure 1, the global features may not be the optimal features for the multi-class classification problem, and using the class-specific features of each binary classification problem obtained by the decomposition strategy would be easier to distinguish the positive class from the negative class. Therefore, if the effects of the binary classifiers can be improved by the class-specific features, then the classification result of the multi-class classification problem can be improved as well.

Using gene expression profile data for detecting cancers is a kind of multi-class classification and these data encounter the following problems: a large number of highly correlated features and limited data samples. Considering the characteristics of expression profile data and class-specific feature selection method, an idea of embedding the elastic net was proposed in the probabilistic SVM to improve the effect of cancer detection, and the working principle is discussed as follows. For the multi-classification problem, the OVA strategy was used to decompose the initial problem into multiple binary classification problems. For each binary classification problem, the elastic net is built using the class label as the dependent variable and features as independent variables. A series of λ are taken into the elastic net to generate coefficients with different degree of sparsity indicating candidate class-specific feature sets. Then these candidate class-specific feature sets are fed into binary classifiers to test their effectiveness and the one achieves the highest accuracy with cross-validation (CV) is chosen as the optimal class-specific feature set. In this way, multiple class-specific feature sets can be obtained for the multi-class classification problem.

**Figure 2:**
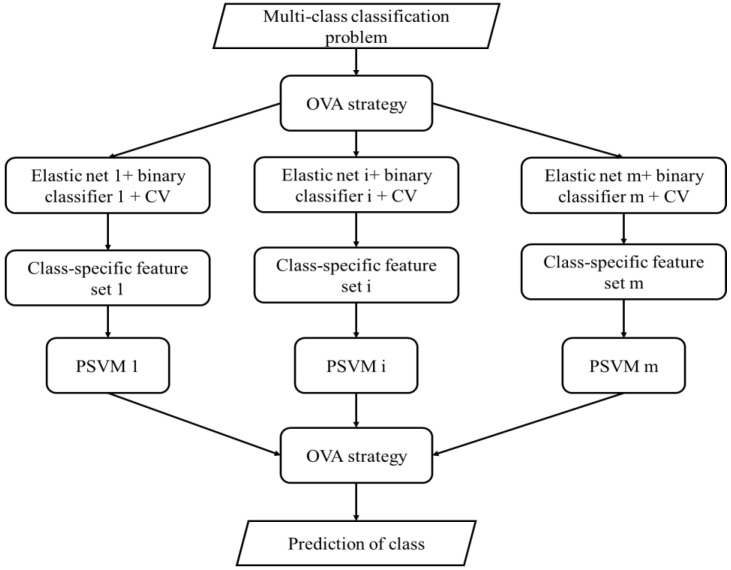
The flowchart of the proposed method.

Using the OVA strategy to deal with the multi-class classification problem, the predicted class is taken from the binary classifier with maximum confidence:

(7) $$\mathrm{class}=\arg\underset{i=1,\cdots ,m}{\max}f_{i}$$

where $f_{i}$ is the estimated confidence measure of the i-th classifier. Using global features, if the SVM classifier is chosen, then the $f_{i}$ can be measured as the decision output of the SVM classifier. However, using the class-specific features, the decision outputs of the SVM classifiers based on different features are not comparable. In order to solve this problem, it is to choose the PSVM classifier, which changes the decision output of the SVM classifier into probability, and this algorithm was named as EPSVM. The probabilistic outputs of the PSVM classifiers normalize the decision outputs of the SVM classifiers, which make the decision outputs of SVM classifiers comparable regardless of whether they are fed with the same features or not. The flowchart of using class-specific features to solve the multi-class classification problem with elastic net and EPSVM is given in Figure 2.

## 4. Experiments

The simulation data and gene expression profile data were provided here, to verify the effect of proposed EPSVM. The SVM classifier combined with global feature selection methods, such as SVM-RFE and Relieff is used to evaluate the performance of EPSVM. The SVM and PSVM are taken from LIBSVM [38]. All calculations of these experiments were performed in MATLAB 2016.

### 4.1. Datasets

#### 4.1.1. Simulation Data

The performance of the proposed algorithm is related to the number of classes. Therefore, the simulation dataset is designed to gradually increase the number of classes to continuously show the advantage of the proposed algorithm. The simulation dataset includes nine data subsets produced in the following way. The data i (1≤i≤9) contains i+2 classes and each class contains 30 samples. The features of data i are produced by two normal distributions, which are $F_{1}\sim N\left(2,1\right)$ and $F_{2}\sim N\left(0,1\right)$. For the class l (1≤l≤i+2), the features from [1+(l−1)×20] to [l×20] are produced by $F_{1}\sim N\left(2,1\right)$ and the rest features from [1] to [20×(i+2)] are produced by $F_{2}\sim N\left(0,1\right)$. The features from [20×(i+2)+1] to [40×(i+2)] are the repeat of features from [1] to [20×(i+2)]. The feature dimension of data i is 40×(i+2). All the features are added with random noise $F_{3}\sim N\left(0,2\right)$. Taking data 1 for example, its structure is shown in Table 1.

#### 4.1.2. Platelets Data for Cancer Detection

**Figure 3:**
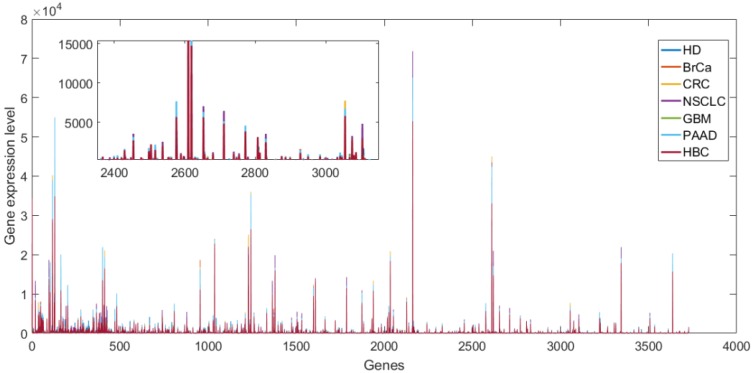
The average gene expression levels of each class in dataset 15.

The gene expression profile data of platelets was measured by RNA-Sequencing technology [39,40], which is a second-generation sequencing technology and has become an important tool in gene expression research. The platelet data was downloaded from the NCBI Gene Expression Omnibus (GEO) database with the accession number GSE68086. The downloaded platelet data has already been pre-processed and can be directly used for testing the performance of the classification algorithm. If readers are interested in the acquisition, actual measurement, and pretreatment methods of platelet data can read the Reference [41] The platelets data contains healthy donors (HD) and patients with various types of cancer including pancreatic cancer (PAAD), non-small cell lung carcinoma (NSCLC), hepatobiliary cancer (HBC), colorectal cancer (CRC), glioblastoma (GBM), and breast cancer (BrCa) with expression levels of 57,737 genes. The platelet data was divided into six data with different classes, and then for the obtained data, the ANOVA test was used to filter the irrelevant features (*p* > 0.001) [42,43]. Table 2 provides the details of the platelet data after processing. Besides, the average gene expression levels of each class in dataset 15 are shown in Figure 3, and it can be seen that the gene expression profile data is seriously overlapped.

#### 4.1.3. Microarray Data for Cancer Detection

The microarray technology [44] is widely used in molecular biology research. Several microarray data have been used to analyze differential gene expression on the transcription level between normal cells and cancer cells [45]. The microarray data were downloaded from http://www.gems-system.org/ and their information is shown in Table 3.

### 4.2. Parameter Settings

For each data, two-thirds of the samples in each class are randomly chosen to form the training set and the rest samples are reserved for independent testing. In the training process, the parameters are set as follows. We adopt the Gaussian kernel for the classifiers of SVM and PSVM and use the grid search method to find the best parameter setting of (c,g) in the space $\left\{2^{-10},2^{-9},\cdots ,2^{9},2^{10}\right\}\times \left\{2^{-15},2^{-9},\cdots ,2^{9},10^{15}\right\}$. The candidate class-specific feature subsets of EPSVM are evaluated by the PSVM classifier using 5-fold cross-validation. The number of nearest neighbors in Relieff is set to 5. The kernel type of SVM-RFE is set to the linear kernel. For the ranking features produced by the global feature selection method (SVM-RFE or Relief), the candidate feature subsets are evaluated by the SVM classifier using 5-fold cross-validation. The rest parameters are the defaults. In the testing process, considering the class imbalance problem of the data, parameter of not error rate (*NER*) is taken as an evaluation index, which is defined as

(8) $$NER_{i}=\frac{SE_{i}+SP_{i}}{2}$$

(9) $$NER=\frac{\sum_{i=1}^{m}NER_{i}}{m},$$

where $NER_{i}$, $SE_{i}$, and $SP_{i}$ are the not error rate, sensitivity [46] and specificity [47] of the *i*-th binary classification problem obtained by the OVA strategy and NER is the not error rate of the multi-class classification problem. This process is intended to repeat 30 times and the average NER is taken as the evaluation index.

## 5. Results and Discussion

### 5.1. Simulation Experiment

According to the generating method of simulation data, the features of the data i (1≤i≤9) were divided into i+2 regions and each region includes 20 features. The features in the same region are independent and the features that i+2 regions apart are corrected. So the simulation data are collinear, high dimensional and small sample. The features that can separate class l (1≤l≤i+2) in data i from the rest classes are located in two regions. The first region includes features from [1+(l−1)×20] to [l×20] and the second region includes features from [1+(l+i+1)×20] to [1+(l+i+2)×20] and the features of these two regions are correlated.

**Figure 4:**
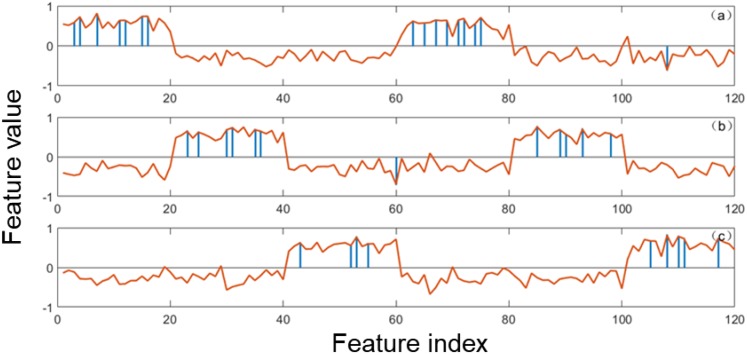
The selected class-specific features of dataset 1 by EPSVM: (**a**) treating class 1 as the positive class; (**b**) treating class 2 as the positive class; (**c**) treating class 3 as the positive class. Indicates the selected feature and indicates the average feature vector of one class.

**Figure 5:**
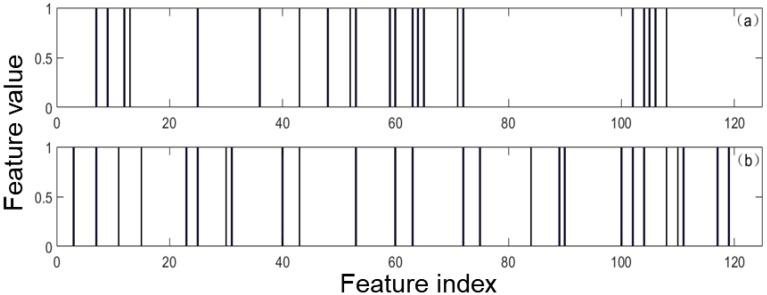
The select global features: (**a**) support vector machine recursive feature elimination (SVM-RFE); (**b**) Relieff. Indicates the selected feature.

In this work, it uses one experiment of data 1 to show the feature selection results of the three methods. Figure 4 shows that EPSVM selects accurately the class-specific features in the two regions for each class. Figure 5 shows that the selected global features by the feature methods of SVM-RFE and Relieff, and one can see that these selected features are located in all regions.

The classification results of the simulation data are shown in Table 4, which shows that the performance of the EPSVM classifier performs better than the other two methods as increasing the number of classes. This is because the class-specific features for the binary classification problems are different. Therefore, the global features are suboptimal for binary classification problems. The differences between the global feature and the class-specific features of the binary classification problem increased as the number of the class is increased. For the data with fewer classes, such as data 1 to 5, EPSVM is a little better than the other methods while for the data with more classes, such as data 6 to 9, EPSVM is much better than the other methods. In thinking further of the OVA strategy, one can find that only the features reflecting the differences between positive class and negative class are effective for the obtained binary classification problem. Therefore, the useless features that only reflect the differences within the negative class, are automatically removed by the class-specific feature selection method, which makes the feature more compact than the global features.

### 5.2. Cancer Detection with the Gene Expression Profile

The performance of the elastic net for the binary classification problem with gene expression profile data is a key factor for the success of the proposed method. We choose the dataset 10 (HD+BrCa) to show the effect of the elastic net comparing with the other two feature selection methods in detail. According to the ranking of p-values, we gradually increase the dimensionality of the feature dimension to test the performance of the three feature selection methods. Table 5 shows that the results produced by the three feature selection methods are comparable regarding improving the generalization performance of classifiers, but the elastic net is more stable than Relieff and SVM-RFE when the size of feature search space changes considerably.

Table 5 shows that the elastic net is well suitable for feature selection in binary classification problem of platelet data. We will further investigate the effect of the elastic net for feature selection in the multi-class classification problem of platelet data. In the multiple classification problems, the feature selection and classifier design are more complicated. The experiment results of the three methods for the platelet data with the different number of classes are shown in Table 6. We can see that for the platelet data with fewer classes, such as data 11 to 13, the performance of EPSVM is slightly better than Relieff+SVM and SVM-RFE+SVM. However, for the platelets data with more classes, such as data 14 to 15, EPSVM shows a relatively great advantage comparing to other methods. Therefore, the proposed method works well for platelets data.

**Figure 6:**
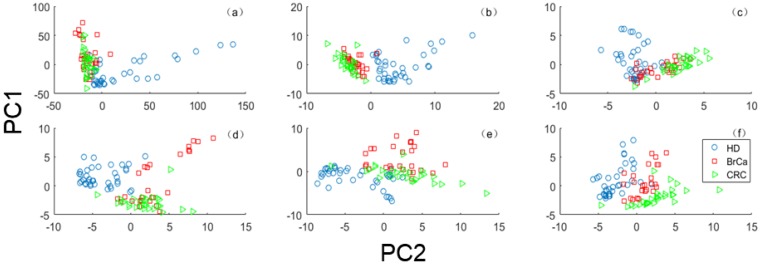
Principal component scores scatter plot (PC 1 × PC 2) of Dataset 11 with different features: (**a**) raw features; (**b**) global features selected by SVM-RFE; (**c**) global features selected by Relieff; (**d**) class-specific features treating healthy donors (HD) class as positive class; (**e**) class-specific features treating BrCa class as positive class; (**f**) class-specific features treating colorectal cancer (CRC) class as positive class.

We plot the first two principal component scores of dataset 11 with raw features and selected features in one experiment in Figure 6. One can see that the cancer classes (BrCa class and CRC class) are strongly overlapping and partially overlap with the HD class. Therefore, the key difficulty of platelet data relies on how to improve the classification result among the cancer classes. According to Figure 6, we can see that the class-specific features determined by EPSVM make the class separate from other classes more clearly than the global features, especially for the cancer classes.

The OVA strategy comes with an inherent flaw that the positive class and negative class are imbalanced [45,46]. Table 7 and Table 8 show the SN and SP of the three methods for dataset 10 and data 14 respectively. We can see that EPSVM shows an obvious advantage in SN when the data including more classes. For the data with more classes, the positive class and negative class are more unbalanced using the OVA strategy. It seems that the EPSVM using class-specific features is more resistant to the impact of class-imbalance than the traditional method using global features under the OVA strategy for the multi-class classification problem.

**Figure 7:**
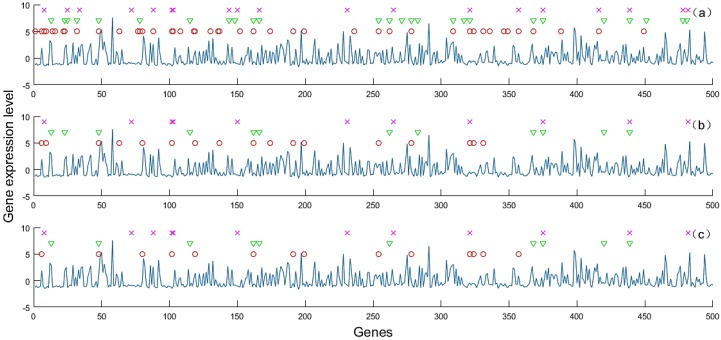
Feature selection results of proposed method with different α in one experiment for Dataset 11 (only the top 500 features are shown), where ×, ▽, ○ indicate the selected class-specific features respectively, and – indicates the gene expression levels of one sample: (**a**) α=0.1, the total number of selected feature is 477; (**b**) α=0.5, the total number of selected feature is 272; (**c**) α=0.9, the total number of selected feature is 253.

Besides, the penalty parameter α∈(0,1) of the elastic net determines how much weight should be given to either ${\left|\beta \right|}_{1}$ or ${\left\|\beta \right\|}^{2}$. It is known that an elastic net with α closer to 1 performs similarly with the LASSO. Especially, the feature selection results of the proposed method for Data 10 in one experiment is shown in Figure 7. We can see that the proposed method with smaller α selects fewer features, but the differences between them are small compared to the feature dimension of the data. Therefore, according to Table 5 and Figure 7, we can find that the proposed method has good robustness towards to α.

We also use microarray data to further demonstrate the validity of the proposed method. Table 9 shows that EPSVM achieves the best performance in most microarray data. Especially for the microarray data including more classes, such as the data of Brain_Tumor1, Tumors_9, and Tumors11, EPSVM is significantly better than the other two methods.

In general, the gene expression profile data has the problems of high dimension, small sample and collinearity. With more classes, the classification problem becomes harder to be dealt with by the global features. The proposed EPSVM can achieve better effects of cancer detection with gene expression profile data than the traditional method based on global feature selection methods.

## 6. Conclusions

In this work, a novel multi-class classification method was proposed to detect cancer diseases with the gene expression profile data, which has the problem of high dimension, small sample, and collinearity. In the proposed method, the class-specific feature selection, OVA strategy and probabilistic support vector machine were innovatively combined to enhance the effect of cancer detection with gene expression profile data. The experiment results show that the proposed method can achieve better classification results than conventional methods based on global feature selection methods. So that the proposed method is suitable for the problem of cancer detection with gene expression profile data.

In general, the proposed method is suitable for multi-class classification problems, where the class-feature set of each class is significantly different from others. Besides, the idea behind the proposed method in this paper can be easily extended to the combination of other feature selection methods and classifiers, which is flexibly in solving the multi-feature and multi-class classification problems encountered in practice. However, for the adopted classifiers, how to normalize the outputs of classifiers with different feature subsets is a key problem. The following work will further study the effect of different normalization methods on the class-feature based multi-class classification method.

## Figures and Tables

**Table 1:** Structure of dataset 1.

Classes	Features from [1] to [60]			Features from [61] to [120]		
	1 to 20	21 to 40	41 to 60	61 to 80	81 to 100	101 to 120
Class 1	$F_{1}+F_{3}$	$F_{2}+F_{3}$	$F_{2}+F_{3}$	$F_{1}+F_{3}$	$F_{2}+F_{3}$	$F_{2}+F_{3}$
Class 2	$F_{2}+F_{3}$	$F_{1}+F_{3}$	$F_{2}+F_{3}$	$F_{2}+F_{3}$	$F_{1}+F_{3}$	$F_{2}+F_{3}$
Class 3	$F_{2}+F_{3}$	$F_{2}+F_{3}$	$F_{1}+F_{3}$	$F_{2}+F_{3}$	$F_{2}+F_{3}$	$F_{1}+F_{3}$

**Table 2:** Descriptions of the platelets data.

Data	No. of Classes	No. of Features	No. of Samples
Data 10	HD+BrCa	1809	94
Data 11	HD+BrCa+CRC	3200	136
Data 12	HD+BrCa+CRC+NSCLC	3398	196
Data 13	HD+BrCa+CRC+NSCLC+GBM	3575	236
Data 14	HD+BrCa+CRC+NSCLC+GBM+PAAD	3876	271
Data 15	HD+BrCa+CRC+NSCLC+GBM+PAAD+HBC	3728	285

Note: The sizes for each class, including HD, BrCa, CRC, NSCLC, GBM, PAAD, HBC are 55, 39, 42, 60, 40, 35, 14, respectively.

**Table 3:** Descriptions of microarray data for cancer detection.

Data	No. of Class	No. of Features	No. of Samples
Leukemia1	3	5327	72
Leukemia2	3	11,225	72
Small Round Blue Cells Tumor (SRBCT)	4	83	2308
Brain_Tumor2	4	50	10,367
Brain_Tumor1	5	90	5920
Tumors_9	9	60	5726
Tumors11	11	12,534	174

**Table 4:** *NER* of simulation data.

Simulation Data	EPSVM	SVM-REF+SVM	Relieff + SVM
Data 1	1.0000	0.9344	0.9750
Data 2	0.9896	0.9792	0.9854
Data 3	0.9859	0.9734	0.9594
Data 4	0.9825	0.9738	0.9362
Data 5	0.9896	0.9646	0.9333
Data 6	0.9920	0.9268	0.9196
Data 7	0.9883	0.9148	0.8914
Data 8	0.9806	0.8896	0.8799
Data 9	0.9731	0.8544	0.8456

**Table 5:** *NER* and feature selection results for dataset 10 (HD+BrCa).

Ranking Features	EPSVM (α = 0.5)		Relieff + SVM		SVM-RFE + SVM	
	No. of Selected Features	Mean *NER*	No. of Selected Features	Mean *NER*	No. of Selected Features	Mean *NER*
100	46.1000	0.9294	38.7500	0.9268	15.0000	0.9222
200	52.3000	0.9464	67.5000	0.9519	22.5000	0.9335
400	68.8000	0.9461	140.0000	0.9283	40.0000	0.9200
600	64.6000	0.9409	187.5000	0.9473	60.0000	0.9488
800	81.3000	0.9463	290.0000	0.9415	90.0000	0.9498
1000	78.0000	0.9414	162.5000	0.9524	100.0000	0.9312
1200	80.3000	0.9646	240.0000	0.9424	120.0000	0.9623
1400	85.4000	0.9422	332.5000	0.9243	140.0000	0.9681
1600	79.4000	0.9385	200.0000	0.9472	160.0000	0.9372
1800	84.2000	0.9556	292.5000	0.9280	180.0000	0.9488

**Table 6:** The mean *NER* of different methods for platelet data.

Methods		Dataset 11	Dataset 12	Dataset 13	Dataset 14	Dataset 15
EPSVM	(α = 0.1)	0.8928	0.8216	0.8153	0.8067	0.8133
	(α = 0.5)	0.8758	0.8203	0.8184	0.7943	0.8014
	(α = 0.9)	0.9035	0.8391	0.8269	0.8030	0.7904
SVM-RFE+SVM		0.8624	0.7996	0.7947	0.7432	0.7599
Relieff+SVM		0.8708	0.7386	0.7980	0.7346	0.7603

**Table 7:** SN and SP of dataset 10 for each class (α = 0.5).

Class	SN			SP		
	EPSVM	SVM-REF + SVM	Relieff + SVM	EPSVM	SVM-REF + SVM	Relieff + SVM
HD	0.9074	0.8333	0.9074	1.0000	0.9877	0.9753
BrCa	0.7949	0.8205	0.8718	0.8958	0.8438	0.8333
CRC	0.7857	0.7857	0.6905	0.8710	0.9032	0.9462

**Table 8:** SN and SP of dataset 14 for each class (α = 0.5).

Class	SN			SP		
	EPSVM	SVM-REF + SVM	Relieff + SVM	EPSVM	SVM-REF + SVM	Relieff + SVM
HD	0.8889	0.9444	0.8667	0.9688	0.9455	0.9610
BrCa	0.5692	0.4462	0.4769	0.9244	0.9098	0.9098
CRC	0.7500	0.5900	0.6400	0.9040	0.8987	0.8693
NSCLC	0.8462	0.7538	0.6923	0.9805	0.9707	0.9659
GBM	0.4833	0.4500	0.5000	0.9687	0.9494	0.9422
PAAD	0.6429	0.5429	0.5571	0.8988	0.8864	0.9136
HBC	0.4000	0.3600	0.3600	0.9933	0.9911	0.9889

**Table 9:** *NER* of microarray data for tumor detection.

Data	EPSVM	SVM-RFE+SVM	Relieff+SVM
Leukemia1	0.9593	0.9333	0.9445
Leukemia2	0.9852	0.8987	0.9589
SRBCT	0.9958	0.9644	0.9844
Brain_Tumor2	0.7581	0.7590	0.7979
Brain_Tumor1	0.8293	0.7244	0.7862
Tumors_9	0.8293	0.7244	0.7862
Tumors11	0.9595	0.9016	0.9182
